# Metabolism of the dual FLT-3/Aurora kinase inhibitor CCT241736 in preclinical and human *in vitro* models: Implication for the choice of toxicology species

**DOI:** 10.1016/j.ejps.2019.04.004

**Published:** 2019-11-01

**Authors:** Francesca L. Wood, Sam Shepherd, Angela Hayes, Manjuan Liu, Katia Grira, Yi Mok, Butrus Atrash, Amir Faisal, Vassilios Bavetsias, Spiros Linardopoulos, Julian Blagg, Florence I. Raynaud

**Affiliations:** Cancer Research UK Cancer Therapeutics Unit, The Institute of Cancer Research, London, United Kingdom

**Keywords:** *In vitro* metabolism, Metabolite identification, Reaction phenotyping, Toxicology species

## Abstract

CCT241736 is a dual fms-like tyrosine kinase 3 (FLT3)/Aurora kinase inhibitor in development for the treatment of acute myeloid leukaemia. The successful development of any new drug relies on adequate safety testing including preclinical toxicology studies. Selection of an appropriate preclinical species requires a thorough understanding of the compound's metabolic clearance and pathways, as well as other pharmacokinetic and pharmacodynamic considerations. In addition, elucidation of the metabolising enzymes in human facilitates improved clinical prediction based on population pharmacokinetics and can inform drug-drug interaction studies. Intrinsic clearance (CL_int_) determination and metabolite profiling of CCT241736 in human and four preclinical species (dog, minipig, rat and mouse) was undertaken in cryopreserved hepatocytes and liver microsomes. Recombinant human cytochrome P450 bactosomes (rCYP) were utilised to provide reaction phenotyping data and support prediction of metabolic pathways. CCT241736 exhibited low CL_int_ in both hepatocytes and liver microsomes of human, dog, minipig and rat, but considerably higher CL_int_ in mouse. CYP3A4 and CYP3A5 were identified as the major enzymes responsible for biotransformation of CCT241736 in human, exclusively forming five out of seven metabolites. Minipig showed greatest similarity to human with regard to both overall metabolic profile and abundance of specific metabolites relative to parent compound, and is therefore proposed as the most appropriate toxicological species. The greatest disparity was observed between human and dog. Based on metabolic profile, either mouse or rat is a suitable rodent species for toxicology studies.

## Graphical abstract

**Figure:**
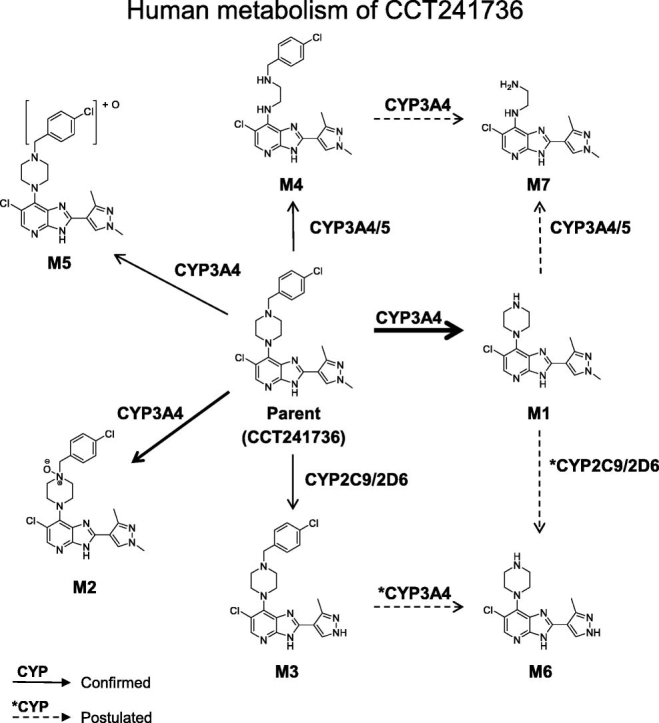

## 1 Introduction

The family of highly homologous serine/threonine kinases known as Aurora (A, B and C) is fundamental in cell mitosis, each isoform regulating discrete yet pivotal processes necessary for successful cell division. Aurora-A, predominantly localised to centrosomes and attached microtubules, has important functions in centrosome maturation as well as spindle assembly and orientation during metaphase I (Carmena and Earnshaw, 2003). Aurora-B forms a complex with two additional proteins at the centromere and is involved in governing chromosome condensation and orientation, interactions between the kinetochore and cytoskeleton, and cytokinesis (Carmena and Earnshaw, 2003). Less is known about the role of Aurora-C, which is thought only to be expressed the testis, thyroid, placenta and gametes undergoing meiotic division (Bernard et al., 1998; Carmena and Earnshaw, 2003; Kimura et al., 1999; Ulisse et al., 2006; Yang et al., 2010). Overexpression of Aurora-A (Bischoff et al., 1998; Gritsko et al., 2003; Reichardt et al., 2003; Tanaka et al., 1999; Zhou et al., 1998)) and Aurora-B (Araki et al., 2004; Chieffi et al., 2004; Sorrentino et al., 2005) has been reported across a number of human malignancies including glioma, breast, ovarian, pancreatic and colon cancer. Inhibition of either Aurora-A or Aurora-B results in cell death, mediated *via* different mechanisms related to the isoforms' distinct functions (Kaestner et al., 2009). Development of Aurora kinase inhibitors therefore became an attractive pharmaceutical strategy with the goal to treat both solid tumours and hematologic cancers, the latter of which was successful, particularly in acute myeloid leukaemia (AML) (Bavetsias and Linardopoulos, 2015). Although single isoform specific Aurora inhibitors were developed, pan-Aurora inhibitors and even those with an additional anti-cancer pharmacology, such as fms-like tyrosine kinase 3 (FLT3) inhibition were also pursued (Bavetsias and Linardopoulos, 2015; Pollard and Mortimore, 2009).

FLT3 is a tyrosine kinase receptor of the platelet-derived growth factor receptor (PDGF-R) sub-family. Both overexpression and activating mutations, including internal tandem duplication (FLT3-ITD), of this receptor have been identified in human leukaemic cell lines (Levis and Small, 2003) and are associated with a poor prognosis in AML (Moore et al., 2010), highlighting it as a potential pharmacological target. However, single agent FLT3 inhibitors developed for the treatment of AML, such as sorafenib and AC220, rapidly elicited acquired resistance (Knapper, 2011). Simultaneous targeting of the Aurora kinases and FLT3 was therefore hypothesised, and subsequently demonstrated to be a promising approach to AML therapy (Bavetsias et al., 2012).

6-Chloro-7-(4-(4-chlorobenzyl)piperazin-1-yl)-2-(1,3-dimethyl-1*H*pyrazol-4-yl)-3*H*-imidazo[4,5-*b*] pyridine (CCT241736, also referred to as Compound **27e** in Bavetsias et al. (2012)), is a dual FLT3 and Aurora kinase inhibitor in pre-clinical development for the treatment of AML. CCT241736 exhibits potent inhibition of FLT3 (K_d_ = 6.2 nM), FLT3-ITD (K_d_ = 38 nM) and Aurora-A and Aurora-B kinases (K_d_ = 7.5 nM and 48 nM, respectively) (Bavetsias et al., 2012). CCT241736 exhibited anti-proliferative activity against HCT116 (human colon carcinoma), MOLM-13 and MV4-11 (human FLT3-ITD positive AML) cell lines, as well as strong inhibition of tumour growth in an MV4-11 xenograft mouse model (Bavetsias et al., 2012). In a 4-day mouse PK/PD study, administration of CCT241736 p.o. at 50 and 100 mg/kg b.i.d. resulted in biomarker modulation (histone H-3 and STAT5 phosphorylation) consistent with target inhibition (Bavetsias et al., 2012). Relative to its predecessor (Compound **6**, (Bavetsias et al., 2012)), CCT241736 was specifically optimised to reduce hERG blockade (IC_50_ > 25 μM) and *in vitro* microsomal metabolism. Pharmacokinetic studies of CCT241736 in mouse and rat revealed moderate (48 ml/min/kg) and low (4.57 ml/min/kg) clearance, respectively, and high oral bioavailability (79–100%) consistent with reported Caco-2 permeability of 18.6 × 10^−6^ cm/s, with no evidence of efflux (Bavetsias et al., 2012). Additionally, a cytochrome P450 (CYP) inhibition screen showed no significant inhibition of CYP1A2, CYP2A6, CYP2C9, CYP2C19, CYP2D6 or CYP3A4; all IC_50_ values were >10 μM (Bavetsias et al., 2012). Thus, CCT241736 was considered to possess properties suitable for further development.

Prior to administration to humans, a drug candidate must undergo nonclinical safety evaluation, typically animal toxicology studies, which assess drug exposure and associated adverse events. Such studies should, where possible, be undertaken in a species which reflects human risk, preferably with a similar pharmacology, pharmacokinetic and metabolic profile (U.S. Department of Health and Human Services Food and Drug Administration Center for Drug Evaluation and Research (CDER), 2016). As it is not uncommon for the metabolism of a compound to differ across species, both quantitatively and qualitatively, selection should be guided by prior *in vitro* and/or *in vivo* metabolic characterisation. The U.S. Department of Health and Human Services Food and Drug Administration Center for Drug Evaluation and Research (CDER) (2016) guidance suggests that any metabolite which is disproportionately higher in human than the studied animal species should undergo separate safety evaluation, and additionally, that any human metabolite predicted to have >10% of total drug exposure should be characterised and monitored in clinical trials. *In vitro* metabolic profiling can therefore prove to be useful in both appropriate selection of toxicological species and clinical trial design. Furthermore, elucidation of the human enzymes responsible for metabolism of a compound can guide both *in vitro* and clinical drug-drug interaction studies and facilitate improved human prediction based on population pharmacokinetics.

Early in drug discovery, large scale screening of metabolic clearance in liver microsomes is an efficient and cost-effective method by which to support structural drug design through the identification of labile groups and to highlight significant species differences. However, due to their subcellular nature, microsomes provide only a limited perspective of metabolism *in vivo*; xenobiotic biotransformation is dependent on the experimental conditions and exogenously supplied cofactors (typically reduced nicotinamide adenine dinucleotide phosphate (NADPH) and increasingly uridine 5′-diphospho-glucuronic acid (UDPGA)), and is unrestricted by plasma membrane permeability. As prediction to preclinical species and to human becomes more relevant, use of *in vitro* systems of increasing complexity (subcellular fractions to whole cells and even organ- or multiple organ-microfluidic systems) which greater mimic the *in vivo* situation, may be advantageous (Bhatia and Ingber, 2014; De Bartolo et al., 2006). Cryopreserved hepatocytes are often utilised to provide a more comprehensive assessment of metabolism due to their ‘off-the-shelf’ availability across multiple species and reference as the ‘gold standard’ for hepatic metabolic clearance determinations (Hewitt et al., 2007). Their full complement of metabolic enzymes and transporters allow assessment of metabolism, uptake and even biliary clearance when used in the appropriate format, including monolayer- and sandwich-culture, but their cost often precludes use in high-throughput settings. Lastly, fundamental systems may still serve to provide specific information, for example the use of recombinant enzymes for reaction phenotyping. Baculovirus- (and other forms of cDNA-) expressed recombinant human enzymes are a simple system in which specific metabolic pathways may be identified and large quantities of metabolites may be generated for the purposes of structural elucidation. They are infrequently used for prediction of overall metabolic clearance due to the need to assess each isozyme individually.

*In vitro* characterisation of CCT241736 metabolism in human and four preclinical species (dog, minipig, rat and mouse) was undertaken. Intrinsic clearance (CL_int_) determinations were performed in both liver microsomes and cryopreserved hepatocytes and metabolite identification was undertaken across all matrices. Additionally, recombinant human CYP (rCYP) were utilised to identify the enzymes likely to be responsible for metabolism of CCT241736 in human.

## 2 Materials and methods

### 2.1 Materials

Cryopreserved human hepatocytes (mixed gender, 20 donors, lot FHQ), Beagle dog hepatocytes (male, 3 donors, Lot FDY; female, 3 donors, Lot JQI) and liver microsomes (DLM) (male, 3 donors, Lot USY; female, 3 donors, Lot APD), Gottingen minipig hepatocytes (male, 3 donors, Lot YNL; female, 3 donors, Lot DPM) and liver microsomes (PLM) (male, 3 donors, Lot NVB; female, donors unknown, Lot VPI), Sprague Dawley rat hepatocytes (female, 21 donors, Lot PCS) and ICR/CD-1 mouse hepatocytes (female, 54 donors, Lot SQW) were purchased from BioIVT (Frankfurt Am Main, Germany). Hepatocyte thawing medium (*InVitro*GRO HT medium) was also purchased from BioIVT. ICR/CD-1 mouse liver microsomes (MLM) (female, 800 donors, Lot 1310224), Sprague Dawley rat liver microsomes (RLM) (female, 200 donors, Lot 1110040) and human liver microsomes (HLM) (mixed gender, 50 donors, Lot 1410013) were purchased from Sekisui XenoTech, LLC (Kansas City, USA). CYP1A2LR, CYP2B6LR, CYP2C8LR, CYP2C9HR, CYP2C19LR, CYP2D6LR, CYP2E1R, CYP3A4LR and CYP3A5BLR EasyCYP bactosomes (each 1 nmol P450/ml co-expressed with human CYP reductase) and inactive control human rCYP bactosomes were purchased from Cypex Limited (Dundee, UK). Williams' Medium E (WME) was purchased from Scientific Lab Supplies Ltd. (Nottingham, UK). 100 mM potassium phosphate buffer (KPB) adjusted to pH 7.4 was prepared in house. All other compounds and reagents were purchased from Sigma-Aldrich Company Ltd. (Dorset, UK). All solvents were obtained from Greyhound Chromatography and Allied Chemicals (Birkenhead, UK). Test and control compound stocks were prepared as 10 mM or 1 mM solutions in DMSO. Olomoucine (500 nM in methanol) was used as an internal standard throughout.

### 2.2 Storage and thawing of cryopreserved hepatocytes

Cells were stored in the vapour phase of liquid nitrogen and thawed as per the supplier's instructions (rapid thawing at 37 °C, followed by centrifugation at 50 ×*g* for 5 min in thawing medium). Hepatocytes were re-suspended in a small volume of incubation medium (WME buffered with HEPES (final concentration 24 mM) adjusted to pH 7.4). Cell density was estimated using a haemocytometer and light microscope and viability determined according to the trypan blue exclusion method; cells were subsequently diluted to the required concentration in incubation medium.

### 2.3 Incubation of hepatocyte suspensions

Suspended hepatocyte incubations for the purposes of metabolite identification and CL_int_ determination were conducted as described by Wood et al. (2018) in round-bottomed 350 μl 96-well polypropylene plates on a PHMP thermoshaker (Grant Instruments (Cambridge) Ltd. (Shepreth, UK)) at 37 °C and 900 rpm. 60 μl of hepatocytes at twice the desired final concentration (Table 1) was pre-incubated for 5 min under the above conditions; 60 μl of CCT241736 (final concentration 1 μM) in incubation medium was added to initiate the reaction. Individual wells were quenched with 120 μl of methanol containing internal standard over a time course (Table 1) and an aliquot removed for analysis.

**Table 1:** Incubation conditions of CCT241736 in cryopreserved hepatocyte suspensions.

Species	Sex	Hepatocyte density (final) (10^6^ cells/ml)	Sampled incubation time points (min)
Human	Mixed	2.5	0, 20, 40, 60, 90, 120
Dog	Male	1	0, 10, 20, 40, 60, 90, 120, 180
	Female	1	0, 5, 10, 20, 40, 60, 90, 120
Minipig	Male	1	0, 10, 20, 40, 60, 90, 120, 180
	Female	1	0, 2.5, 5, 10, 20, 30, 40, 60
Rat	Female	1	0, 10, 20, 30, 40, 60, 90, 120
Mouse	Female	0.25	0, 10, 20, 30, 40, 60, 90, 120

### 2.4 Incubation of subcellular fractions and recombinant enzymes

All incubations of subcellular fractions and recombinant enzymes were conducted at 37 °C and 800 rpm using a Hamilton Microlab Star liquid handling workstation (Hamilton Robotics (Bonaduz, Switzerland)).

#### 2.4.1 Microsomal incubations

CCT241736 (final concentration 1 μM) was pre-incubated for 10 min in PLM, DLM, MLM, RLM or HLM (final concentration 1 mg/ml prepared in 10 mM phosphate buffered saline (PBS)). Prior to initiation of the reaction by the addition of NADPH (final concentration 1 mM), an aliquot was removed from each incubation and quenched in ice cold methanol containing internal standard (referred to as T_0_). NADPH was subsequently added to this solution for matrix matching. The remaining reaction (with addition of NADPH) was allowed to proceed for 60 min. At 0.5, 5, 15, 30 and 60 min aliquots were sampled and quenched as above. Incubations were conducted in singlicate; control incubations (substituting PBS for NADPH) were conducted simultaneously.

#### 2.4.2 Reaction phenotyping using recombinant CYP

Recombinant CYP (100 pmol P450/ml) and control bactosome (at an equivalent protein concentration) were prepared in 100 mM KPB. CCT241736 (final concentration 1 μM) was pre-incubated for 3 min with each of rCYP1A2, rCYP2B6, rCYP2C8, rCYP2C9, rCYP2C19, rCYP2D6, rCYP2E1, rCYP3A4 and rCYP3A5 and control. The reaction was initiated by the addition of NADPH (final concentration 1 mM) and allowed to proceed for 20 min. At T_0_ (see Section 2.4.1), 0.5, 5, 10, 15 and 20 min, an aliquot was removed from each incubation and quenched in ice cold methanol containing internal standard.

### 2.5 Protein binding studies

The fraction unbound in microsomes and rCYP (fu_mic_ and fu_CYP_ respectively) of CCT241736 was measured using a Rapid Equilibrium Dialysis device (Thermo Fisher Scientific, Loughborough. UK). HLM (1 mg/ml) and control rCYP (100 pmol/ml) containing 1 μM CCT241736 were dialysed against 100 mM KPB for 4 h at 37 °C. Samples were removed, matrix matched and quenched with methanol containing internal standard; further processing is as described in Section 2.6.

### 2.6 Sample preparation and liquid chromatography-mass spectrometry analysis

Terminated hepatocyte incubations were stored in a −20 °C freezer for at least 1 h before further processing. All samples were centrifuged at 3700 rpm at 4 °C using an Eppendorf 5810 R centrifuge (Eppendorf UK Limited, Stevenage, UK) for 30 min to precipitate protein and enable sampling of supernatant prior to appropriate dilution for liquid chromatography-mass spectrometry (LC-MS(/MS)) analysis.

For the purpose of CL_int_ determination, samples were analysed using an Agilent 1290 Infinity ultra high-performance liquid chromatography (UHPLC) system coupled to an Agilent 6520 Quadrupole Time-of-flight mass spectrometer (Agilent Technologies, Stockport, UK). Separation of analytes was achieved using a Phenomenex Kinetex C18 column (2.6 μm, 50 × 2.1 mm) (Phenomenex, Macclesfield, UK) at a temperature of 55 °C and a binary mobile phase gradient at a flow rate of 0.6 ml/min. Initial LC conditions comprised 90% solvent A (0.1% formic acid in water), 10% solvent B (methanol); this was ramped to 100% B at 3 min, held for 1 min and then immediately returned to initial conditions and held for the remaining 2 min of the method. Sample analysis was by electrospray atmospheric pressure ionization combined with full scan acquisition in positive ion mode over a mass range 100–1000 *m*/*z*. The capillary voltage was 4 kV, desolvation gas was 350 °C.

For the purpose of metabolite identification, samples were analysed using a Dionex Ultimate 3000 UHPLC system coupled to a ThermoScientific Q Exactive Plus orbitrap mass spectrometer (Thermo Fisher Scientific Inc., Waltham, USA). Separation of analytes was achieved using an Acquity UPLC HSS T3 column (1.8 μm, 100 × 2.1 mm) (Waters, Elstree, UK) at a temperature of 50 °C and a binary mobile phase gradient at a flow rate of 0.4 ml/min. Initial conditions comprised 10% solvent A (0.1% formic acid in water), 90% solvent B (methanol); this was ramped to 95% A at 12 min, immediately returned to initial conditions and held for the remaining 3 min of the method. The UV absorbance of samples at 230 nm and 260 nm was monitored using a Dionex Ultimate 3000 diode array detector. Sample analysis was by electrospray atmospheric pressure ionization combined with full scan acquisition in positive ion mode over a mass range 80–1200 *m*/*z*. The capillary voltage was 3.5 kV; desolvation gas and capillary temperatures were 450 °C and 275 °C respectively; sheath, aux and sweep gas flow rates were 55, 15 and 3 respectively. Full MS/dd-MS^2^ (full MS scan followed by data dependent MS/MS) and Full MS/AIF (full MS scan followed by all ion fragmentation) workflows were used in combination. Parameters for these workflows are given in Table 1 of the Appendix.

### 2.7 Metabolite identification

Identification of metabolites and elucidation of their proposed structures was undertaken with the aid of the software Compound Discoverer (v2.0.0.303, Thermo Fisher Scientific Inc., Waltham, USA). RAW data files containing accurate mass data and fragmentation spectra generated in the acquisition software Chromeleon (v7.2 SR3 (7553), Thermo Fisher Scientific Inc., Waltham, USA) were input into Compound Discoverer and processed using the targeted workflow described in Fig. 1 of the Appendix. The final time point of a parallel incubation of a different compound was used as a sample blank and any chromatographic peaks identified with the same *m*/*z* and retention time as in this sample were marked as background compounds and therefore not considered as potential metabolites. The Compound Discoverer application combines accurate mass data, isotope pattern matching and predicted biotransformations based on the parent compound's structure to generate a series of expected and unexpected compounds or metabolites. These were manually filtered using a typical criteria of peak area greater than or equal to 5000 counts per sec; compound identified in samples of at least three time points and peak area ratio of compound in T_x_ relative to T_0_ greater than or equal to 2, where T_x_ represents any given time point. Visual inspection of the chromatograms was undertaken to identify analytes exhibiting a change in intensity consistent with that of a metabolite. The fragmentation spectra of these analytes were compared to those of the parent to facilitate structural interpretation of biotransformations.

### 2.8 Computational chemistry

The protein–ligand co-crystal structure of Aurora-A kinase (Protein Data Bank (PDB) 2x6e) (Bavetsias et al., 2010) was prepared for modelling using Protein Preparation Wizard (Maestro v11.2, Schrödinger, LLC: New York, USA, 2017). To predict proposed binding poses of the ligands, Glide (Grid-based Ligand Docking with Energetics) (v7.5, Schrödinger, LLC, New York, USA, 2017) was used for the docking experiments. The receptor grid was defined by a grid box of 30 × 30 × 30 Å with a default inner box (10 × 10 × 10 Å) (Glide v7.5, Schrödinger, LLC, New York, USA, 2017) centred on the crystallized ligand (compound **51** (Bavetsias et al., 2010)) in the Aurora-A binding site in structure PDB 2x6e. Ligands were prepared using LigPrep (v4.2, Schrödinger, LLC: New York, USA, 2017), applying the OPLS_2005 force-field with possible tautomeric and ionization states within pH range 5.0–9.0 generated using Epik ionization settings. Using Glide Extra Precision (XP) settings, flexible docking of ligands was performed unconstrained.

### 2.9 Metabolite isolation and purification

In order to generate sufficient quantity of M1 to be sent for K_d_ determination, multiple rCYP3A4 incubations (equivalent to those described in Section 2.4.2) were conducted in parallel. The supernatant generated by centrifugation (as described in Section 2.5) of quenched samples was pooled and evaporated to dryness using a Biotage V-10 Touch evaporator (Biotage, Uppsala, Sweden). Sample was reconstituted in 5 ml of 1:1 methanol:water and centrifuged at 5000 rpm for 5 min; 4 × 1 ml of the supernatant was transferred to 1.5 ml vials for separation *via* HPLC. Chromatographic separation was performed using an Agilent 1260 Series HPLC (Agilent, Santa Clara, USA) and a ZORBAX Eclipse XDB-C18 column (4.6 × 150 mm, 5 μm) (Agilent, Santa Clara, USA) at a temperature of 30 °C. 50 μl injections were run over a 12 min gradient elution at 10–95% B at a flow rate of 1 ml/min (organic solvent B: methanol, aqueous solvent A: 0.1% formic acid in water). UV–vis spectra were acquired at 210 nm, 254 nm and 280 nm on a 1260 Series diode array detector (Agilent Santa Clara, USA). The fractions were collected using a 1260 Agilent analytical scale fraction collector. Raw data was processed using Agilent Chemstation software (v C.01.05, Agilent, Santa Clara, USA). The same fractions collected from multiple injections were pooled and dried using a Biotage V-10 Touch evaporator. Isolated M1 was confirmed by LC-MS as described in 2.5, 2.6.

#### 2.9.1 NMR

The absolute weight of the purified M1 was determined by quantitative ^1^H NMR (Nuclear Magnetic Resonance) using the Bruker ERETIC2 method.

0.5 ml of methanol‑*d*_4_ was added to the dried M1 fraction collected in Section 2.8. The concentration of the sample was obtained by quantitative ^1^H NMR and the absolute weight of M1 was calculated. M1 was then recovered by evaporating methanol‑*d*_4_ using a Smart Evaporator (Asynt, Iselham, UK). 3.33 mM DMSO stock solution was then prepared according to the absolute weight of M1. The parent compound CCT241736 was used in parallel as a control.

NMR data was collected on a Bruker Avance NEO spectrometer equipped with 600 MHz magnet and 5 mm TCI Cryoprobe (Bruker, Massachusetts, USA). The ^1^H spectrum was referenced to the internal deuterated solvent. The operating frequency for ^1^H was 600 MHz. All NMR data were acquired at the temperature of 298 K. Data was acquired and processed using Bruker Topspin (v4.0, Bruker, Massachusetts, USA). The quantitative ^1^H NMR spectrum was acquired using a Bruker standard 1D zg pulse sequence with 4 scans. The sweep width was 19.8 ppm, and the FID contained 64 k time-domain data points. Relaxation delay was set to 20 s. The quantitative ^1^H NMR spectrum was acquired using an in-house 1D pulse sequence lc1pngppsf2 with 32 scans. The sweep width was 6.2 ppm, and the FID contained 16 k time-domain data points. Relaxation delay was set to 20 s.

### 2.10 K_d_ determination

Purified solid M1 and parent (CCT241736) were used to prepare 1.665 mM DMSO solutions which were sent to DiscoverX Corporation (Freemont, USA) for K_d_ determination against Aurora-A, Aurora-B, FLT3 and FLT3-ITD using the KINOME*scan*™ profiling service. K_d_s were determined using an 11-point 3-fold compound dilution series (top concentration 5 μM) with three DMSO control points.

### 2.11 Data analysis

LC-MS(/MS) data was processed using the relevant associated software; either Agilent MassHunter Quantitative Analysis (v. B.06.00, Agilent Technologies, Stockport, UK) or Chromeleon 7 Chromatography Data System (v. 7.2 SR3 (7553), Thermo Fisher Scientific Inc., Waltham, USA).

*In vitro* data (parent compound LC-MS response) was fitted to a non-linear single exponential model using the software GraphPad Prism (v6.07, GraphPad Software, Inc., La Jolla, USA) to determine the elimination rate constant (k). CL_int_ in hepatocytes and liver microsomes was calculated using Eqs. (1), (2), respectively.

(1) $${\mathrm{CL}}_{\mathrm{int}}\;\left(\mathrm{\mu l}/\min/10^{6}\;\text{cells}\right)=\frac{V\cdot k}{\mathrm{No}.\text{of cells}\;}$$

(2) $${\mathrm{CL}}_{\mathrm{int}}\left(\mathrm{\mu l}/\min/\mathrm{mg}\;\text{microsomal protein}\right)=\frac{V\cdot k}{\mathrm{mg}\;\text{protein}}$$

where CL_int_ is the intrinsic clearance (μl/min/10^6^ cells or μl/min/mg microsomal protein), V is the incubation volume (μl), No. of cells is the number of cells in the incubation/10^6^ for hepatocyte assays and mg protein is the mg microsomal protein in the incubation for microsomal assays.

CL_int_ in rCYP was calculated using Eq. (3) and scaled to μl/min/mg microsomal protein using Eq. (4).

(3) $${\mathrm{CL}}_{\mathrm{int}}\left(\mathrm{\mu l}/\min/\text{pmol}\;P450\right)=\frac{V\cdot k}{\text{pmol protein}}$$

(4) $${\mathrm{CL}}_{\mathrm{int}}\left(\mathrm{\mu l}/\min/\mathrm{mg}\;\text{microsomal protein}\right)={\mathrm{CL}}_{\mathrm{int}}\left(\mathrm{\mu l}/\min/\text{pmol}\;P450\right)\cdot \text{ISEF}\cdot \mathrm{CYP}\;\text{abundance}\;$$

where ISEF is the Intersystem Extrapolation Factor and CYP abundance is amount of each CYP relative to microsomal protein. For rCYP3A4 an ISEF of 1.09 (Certara, 2017) and a CYP abundance of 137 pmol P450/mg microsomal protein (Certara, 2017) were used.

The fraction unbound (f_u_) in HLM/rCYP was calculated using Eq. (5).

(5) $$f_{u}=1/\left(1+\left(\frac{1}{\frac{\mathrm{PAR}\;\text{receiver}}{\mathrm{PAR}\;\text{donor}}}-1\right)\right)$$

where PAR = Peak Area Ratio of analyte/internal standard.

Unbound CL_int_ (CL_int,u_) was calculated using Eq. (6).

(6) $${\mathrm{CL}}_{\mathrm{int},u}={\mathrm{CL}}_{\mathrm{int}}/f_{u}$$

To enable comparison of metabolites across species and systems, semi-quantitative analysis of metabolites based on LC-MS response was performed. % T_0_ parent response (Eq. (7)) describes the abundance of a single metabolite at any given time point relative to the initial abundance of the parent. % total metabolite response (Eq. (8)) describes the abundance of a single metabolite at any given time point as a percentage of the total abundance of parent and all identified metabolites at that same time point.

(7) $$\%T_{0}\;\text{parent response}=\frac{\mathrm{LC}-\mathrm{MS}\;\text{response of metabolite}\;\mathrm{at}\;T_{x}}{\mathrm{LC}-\mathrm{MS}\;\text{response of parent}\;\mathrm{at}\;T_{0}}$$

where T_x_ represents any given time point and T_0_ represents 0 min.

(8) $$\%\text{total metabolite response}=\frac{\mathrm{LC}-\mathrm{MS}\;\text{response of metabolite}\;\mathrm{at}\;T_{x}}{\text{Combined}\;\mathrm{LC}-\mathrm{MS}\;\text{response of parent and}\;\mathrm{all}\;\text{metabolites}\;\mathrm{at}\;T_{x}}$$

## 3 Results

### 3.1 Comparison of CL_int_ of CCT241736 across *in vitro* systems and species

The CL_int_ of CCT241736 was determined in hepatocytes and liver microsomes of human and four preclinical species, namely dog, minipig, rat and mouse (Table 2). In human, rat, dog and minipig hepatocytes the CL_int_ of CCT241736 was low, ranging between <1–10 μl/min/10^6^ cells, but was considerably higher in mouse hepatocytes (91 μl/min/10^6^ cells). Similarly, the microsomal CL_int_ of CCT241736 was highest in mouse (53 μl/min/mg protein); all other species exhibited low microsomal CL_int_ (<10 μl/min/mg protein (Table 2)).

**Table 2:** CL_int_ of CCT241736 in hepatocytes and liver microsomes of human, dog, minipig, rat and mouse.

Species	Sex	Hepatocyte CL_int_ (μl/min/10^6^ cells)	Microsomal CL_int_ (μl/min/mg protein)
Human	Mixed	<1	7.8 ± 1.0
Dog	Male	8.4	7.4 ± 1.4
	Female	3.9	9.5 ± 2.2
Minipig	Male	7.0	5.5 ± 0.9
	Female	3.4	5.1 ± 1.1
Rat	Female	10	2.7 ± 1.8
Mouse	Female	91	53 ± 4.1

### 3.2 Metabolite profiling of CCT241736 *in vitro*

Metabolite profiling of CCT241736 (Fig. 1) was undertaken on all *in vitro* matrices studied; 13 metabolites were identified in addition to the parent compound (*m*/*z*, 456.147; retention time (RT), 7.58 min). Although UV–vis spectra were acquired for each sample, neither parent compound nor metabolites were detectable in any unprocessed matrix due to their low concentration. UV–vis analysis of the concentrated rCYP3A4 incubate (described in Section 2.8) revealed peaks at 260 nm for parent, M1 and M4. It is believed that the other CYP3A4 metabolites were either not generated in sufficient quantity for UV–vis analysis or became unstable during processing. The ratio of metabolite to parent based on UV trace peak area and LC-MS response were equivalent for M1 and M4 suggesting that mass spectrometry signals are adequate representation of concentration for these metabolites. In the absence of UV data for the other 11 metabolites, the assumption was made that LC-MS response was representative of absolute abundance in these instances also.

**Fig. 1:**
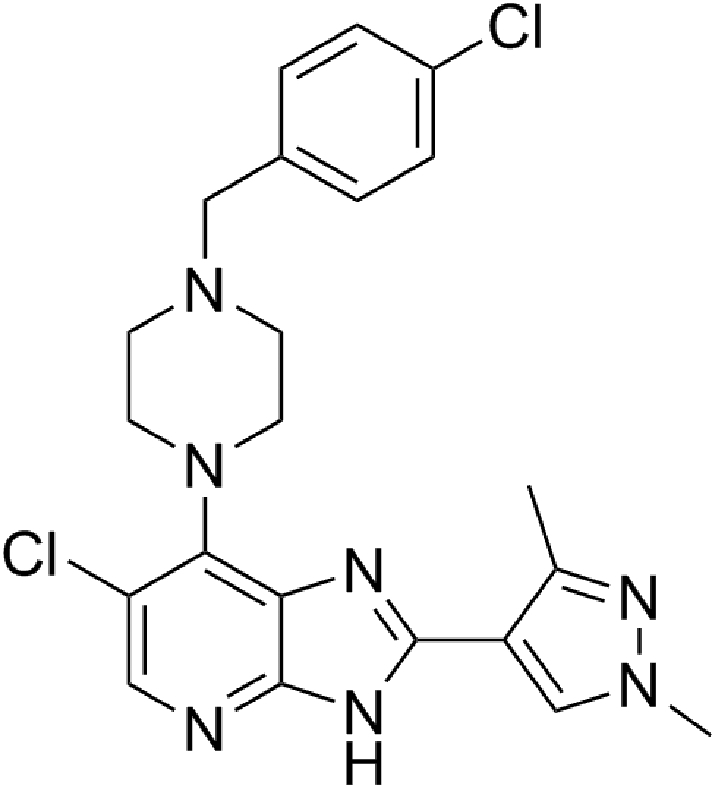
Chemical structure of CCT241736.

**Fig. 2:**
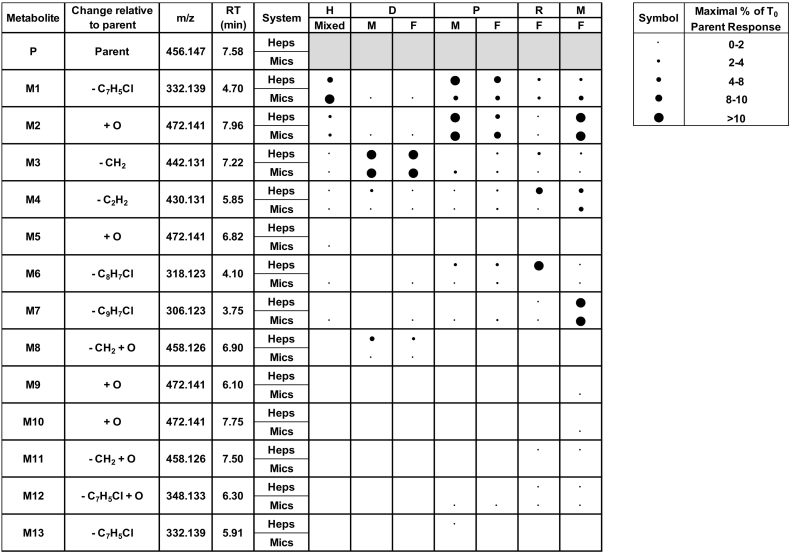
Metabolites of CCT241736 and their maximal abundance (at any time point) relative to parent at T_0_ in hepatocytes and liver microsomes of human, dog, minipig, rat and mouse. RT, retention time; H, human; D, dog; P, minipig; R, rat; M, mouse.

Structural elucidation of metabolites was performed using accurate mass data and fragmentation spectra generated during LC-MS/MS analysis; supporting information is given in Fig. 2A–D of the Appendix. All metabolites were determined to be either oxidations and/or dealkylations of parent. A summary of the identified metabolites and their maximal abundance (relative to parent at T_0_) in each matrix is presented in Fig. 2.

Thirteen metabolites were identified overall; seven of these (five dealkylations and two mono-oxidations) were present in human *in vitro* systems (Fig. 2). At the final sampled time point, the most abundant metabolite in human, M1, represented 9.4% and 10.6% (hepatocytes and HLM respectively) of the parent LC-MS response at T_0_. M2, the second most abundant metabolite in human, gave an LC-MS response in hepatocytes and HLM equivalent to between 2 and 4% of parent LC-MS response at T_0_. Two additional metabolites (M3 and M4) were found in both human hepatocytes and HLM and a further three metabolites (M5, M6 and M7) in HLM only at abundances <2% of T_0_ parent (based on LC-MS response).

Similar to human, M1 was highly abundant in minipig (male and female); however, M2 was of comparably high abundance in this species and therefore increased in minipig relative to human. In addition to those metabolites synonymous with human (M3, M4, M6 and M7), M12 and M13 were identified in PLM (both sexes) and male minipig hepatocytes respectively. In dog, M3 was the major metabolite (maximally >10% of T_0_ parent based on LC-MS/MS response) identified in hepatocytes and microsomes of both sexes, whilst M8, a metabolite found only in dog, showed increased abundance in hepatocytes relative to microsomes (Fig. 2). M1, M2 and M4 were also present in DLM (both sexes); M6 and M7 were identified in female DLM only. In rat, the major metabolite in hepatocytes (M6) was not identified in RLM and the second most abundant metabolite in hepatocytes (M4) (maximally 8–10% of T_0_ parent LC-MS/MS response), represented at most <2% of parent in RLM. Additional minor metabolites identified in rat include M1, M2, M3, M7, M11 and M12. In mouse, M2 and M7 were major metabolites (maximally >10% of T_0_ parent based on LC-MS/MS response) in both hepatocytes and MLM; LC-MS/MS response of M4 and M1 represented between 2 and 8% of initial parent signal. Additional minor metabolites (<2% T_0_ parent response) identified in mouse include M3, M6, M9, M10, M11 and M12 (Fig. 2). Maximal or close to maximal abundances of metabolite were observed at the final sampled time point for all species with the exception of mouse, for which some metabolites notably reached maximal abundance much earlier and subsequently decreased.

**Fig. 3:**
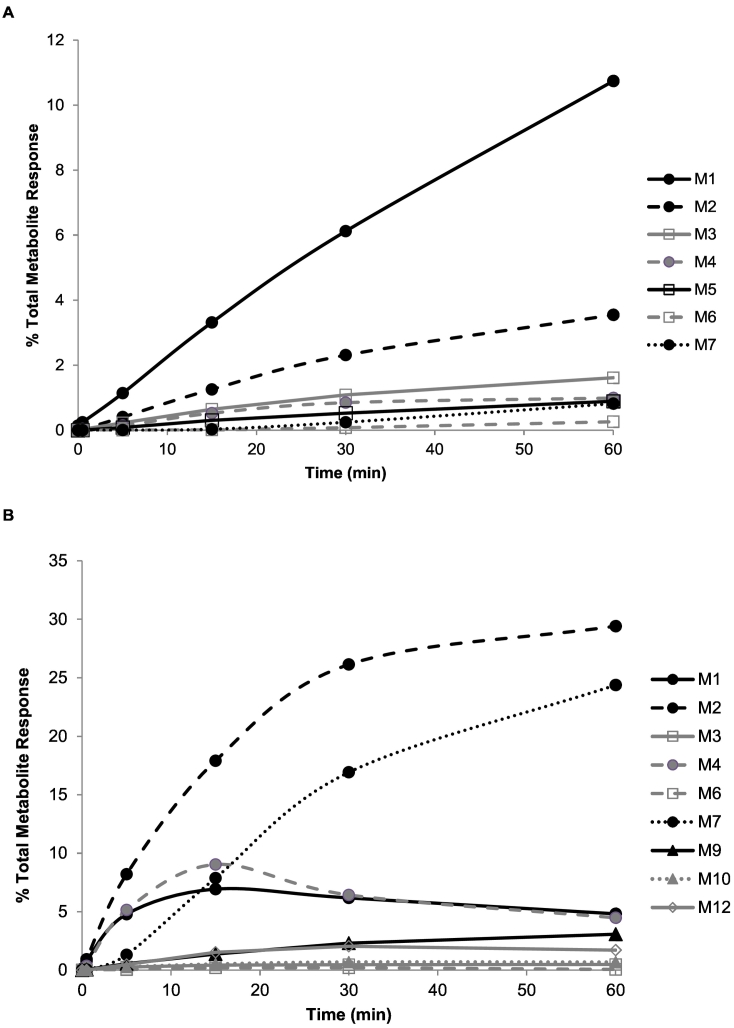
Profile of CCT241736 metabolites following 1 μM incubation in human (A) and mouse (B) liver microsomes of protein concentration 1 mg/ml. Values are percentage of total mass spectrometric response.

Graphical representation of the *in vitro* metabolic profile of CCT241736 across species over time provides further insight into similarities and differences between metabolic pathways which are not obvious from maximal abundance data. The metabolic profiles of CCT241736 and its metabolites (as a percentage of total parent and metabolite response (Eq. (8)) in HLM and MLM are given in Fig. 3A and B respectively. In HLM, M1 (the most abundant metabolite) shows a consistent increase over the 60 min incubation period, however in MLM, M1 shows an initial increase (up to 7% total metabolite response) followed by a slight decrease, potentially indicative of a primary metabolite which is subsequently further transformed. A similar, but more exaggerated profile is also observed for M4 (Fig. 3B). M2 is the most abundant metabolite in MLM, increasing to >25% of total metabolite response within the first 30 min of incubation, but levelling off subsequently despite a continued increase in the abundance of other metabolites (*e.g.* M7). In HLM, M2 is much less abundant, reaching only 3.5% total metabolite response at 60 min incubation, highlighting a species difference in the predominant pathway of CCT241736 metabolism. In HLM, M6 and M7 are undetectable until 15 min incubation, then exhibit a steady increase in abundance, consistent with the profile of a secondary metabolite. M7 follows a similar profile in MLM although at greater abundance than in HLM. The abundance of M6 in MLM is too low to discern a meaningful profile.

### 3.3 Reaction phenotyping of CCT241736 using rCYP

As all biotransformations identified were consistent with Phase I metabolism, reaction phenotyping studies were conducted only in rCYPs. Six of the seven metabolites of CCT241736 (M1-M5 and M7) identified in HLM and human hepatocytes (Fig. 2) were found in single rCYP incubations. Although metabolites were identified in incubations of recombinant CYP3A4, CYP3A5, CYP2C9 and CYP2D6, CL_int_ of CCT241736 could only be accurately determined in rCYP3A4, as depletion of substrate in other rCYP incubations was negligible. The scaled CL_int,u_ of CCT241736 in rCYP3A4 was equivalent to 4078 μl/min/mg microsomal protein (fu_CYP_ = 0.0094). This compares to CL_int,u_ in HLM of 2714 μl/min/mg microsomal protein (fu_mic_ = 0.0018). As well as being the major contributor to the overall CL_int_ of CCT241736, CYP3A4 was demonstrated to be responsible for formation of five out of the seven identified metabolites (M1, M2, M4, M5 and M7). All metabolites identified in rCYP3A4 incubations were also found in incubations of rCYP3A5. M3 was observed in incubations of both recombinant CYP2D6 and CYP2C9, however no distinguishable loss of parent was observed in such incubations, precluding calculation of CL_int_. M6 (identified in HLM) was not observed across any incubations of a single rCYP and is therefore proposed as a secondary metabolite formed *via* sequential metabolism by multiple CYPs.

### 3.4 Docking of M1 to Aurora and FLT3

Molecular docking experiments were conducted to predict the binding of CCT241736 and M1 to Aurora-A and FLT3 kinases. As there are over 100 Aurora-A structures reported in the PDB (Berman et al., 2000), the crystal structure of Aurora-A with a structural analogue of the same series of inhibitor (compound **51** (Bavetsias et al., 2010)) bound (PDB 2x6e) was selected for docking experiments. This kinase structure adopts an ‘active’ conformation that typically binds ‘type-I’ kinase inhibitors, and the protein conformation is compatible with ADP binding. Using this structure, both ligands (CCT241736 and M1) were predicted to adopt a similar binding mode to the bound inhibitor compound **51** (Fig. 5) (Bavetsias et al., 2010). On the contrary, there are only a few FLT3 kinase structures reported in the PDB. They all adopt an ‘inactive’ conformation that typically binds ‘type-II' kinase inhibitors, and the protein conformation is incompatible with ADP binding. Although CCT241736 and M1 could be modelled into an ADP-incompatible binding pocket, it would be illogical to propose such a binding mode prediction given that these compounds would be expected to behave like type-I kinase inhibitors and therefore be ADP-competitive. Hence, a binding pose of these compounds in FLT3 is not reported.

### 3.5 Isolation, purification and activity of M1

Due to the abundance of M1 (maximally approximately 10% of parent) in human hepatocytes and liver microsomes, it was decided to screen M1 for activity against all targets of the parent compound. For this to be possible, μg quantities of the metabolite were required. M1 was successfully isolated and purified using the methods described in 2.9, 2.9.1 and was sent to DiscoverX Corporation for selected K_d_ determinations. M1 was found to at least retain activity against FLT3, although potency against FLT3-ITD, Aurora-A and Aurora-B was reduced compared to parent compound CCT241736 (Table 3).

**Table 3:** Activity of CCT241736 and M1 against FLT3, FLT3-ITD, Aurora-A and Aurora-B.

Compound	K_d_ (nM)			
	FLT3	FLT3-ITD	Aurora-A	Aurora-B
CCT241736^a^	6.2	38	7.5	48
CCT241736	26	34	9.8	64
M1	7.9	82	33	130

a Data taken from Bavetsias et al. (2012).

## 4 Discussion

*In vitro* assessment of the metabolism of CCT241736 was undertaken in human and four pre-clinical species (dog, minipig, rat and mouse) with the aim to establish which species would be most suitable for toxicology studies.

CCT241736 exhibited low CL_int_ in hepatocytes and liver microsomes of human, dog, minipig and rat, not exceeding 10 μl/min/10^6^ cells and 10 μl/min/mg protein respectively. The CL_int_ of this compound in mouse hepatocytes and liver microsomes was considerably higher at 91 μl/min/10^6^ cells and 53 μl/min/mg protein respectively. However, as it is not uncommon for the CL_int_ of a compound to be greater in smaller mammalian species than in human (Naritomi et al., 2001; Nishimuta et al., 2013; Sakai et al., 2014), the overall metabolic profile, together with pharmacokinetics and pharmacodynamics, bears greater influence in selection of a toxicology species.

Metabolite identification was performed for all studied matrices. Although UV spectra were acquired, the low assay substrate concentration and turnover precluded metabolite quantification by this method. Instead, semi-quantitative analysis of metabolites based on LC-MS response relative to that of the parent was performed, with the obvious caveat to data interpretation being the assumption that all metabolites share the same ionization efficiency as the parent compound. Although this may be considered unlikely, the ratio of M1 to parent in the concentrated rCYP3A4 incubate was the same whether based on LC-MS response or UV absorbance at 260 nm. This lends support to the reported relative abundances of each metabolite and the conclusion that M1 is the most abundant metabolite in human *in vitro* systems.

Of the two larger mammalian species, minipig showed greatest similarity to human with regard to both overall metabolic profile and maximal abundance of specific metabolites relative to parent (Fig. 2). Dog was considered to be a less appropriate toxicology model due to the increased abundance of M3 (relative to parent) and the presence of M8, a metabolite uniquely identified in dog, also at relatively high levels (up to 8% of initial parent response).

In rodents, 6 out of 7 metabolites found in human were also observed in both rat and mouse, with additional low abundance metabolites identified in both species. Although there appears to be considerable dissimilarities in the abundance of specific metabolites of CCT241736 in rodents and human (Fig. 2), at least for mouse, these may reflect the higher CL_int_ in this species, rather than differences in the predominance of certain metabolic routes. For example, M2 is a primary metabolite which is not believed to undergo further biotransformation; the increased abundance of M2 in mouse relative to human is therefore consistent with increased overall metabolism of the parent compound. The same principle could be argued for M6 in rat hepatocytes; its apparent absence in RLM and human hepatocytes representative of the reduced turnover of parent in these systems. Overall both mouse and rat *in vitro* systems provide good representation of human metabolites observed *in vitro*.

M5, a mono-oxidation, was the only human metabolite not to be identified in any of the investigated pre-clinical species. However, as it is only present in HLM at very low abundance, it is not considered a significant cause for concern.

**Fig. 4:**
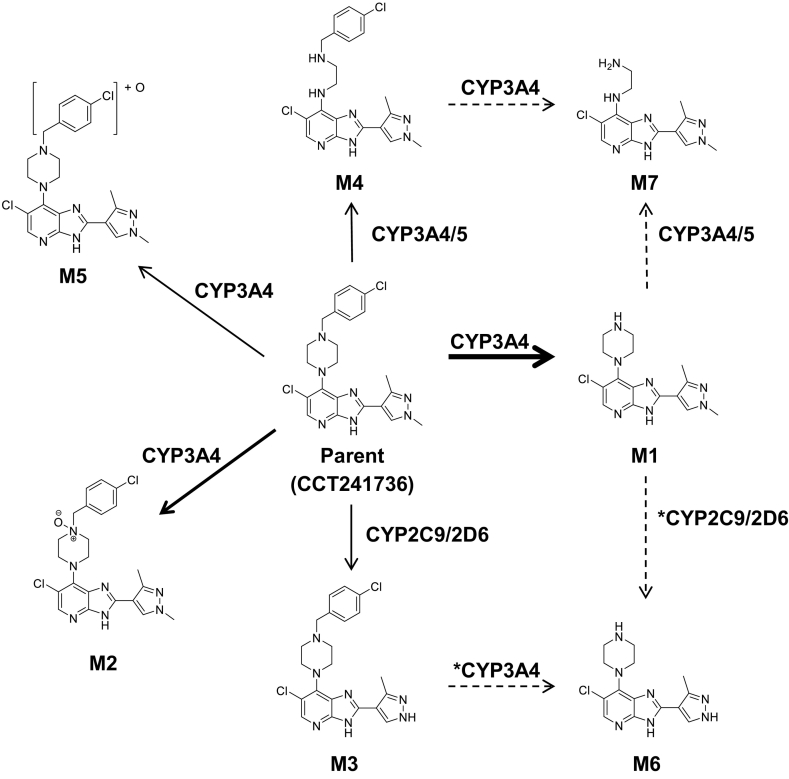
Proposed *in vitro* metabolic biotransformation pathway for CCT241736 in human. Bold arrows represent predominance of these metabolic pathways. Dotted arrows and asterisked enzymes represent postulated secondary metabolic pathways based on known metabolite structures and primary biotransformations.

Notably, all metabolites identified in human hepatocytes were also present in HLM (supplemented with NADPH only), indicating that all detectable metabolism of CCT241736 was mediated by CYPs or microsomal enzymes which do not require exogenous cofactors. Reaction phenotyping of CCT241736 was therefore undertaken using recombinant cytochrome P450 bactosomes, of which those containing the nine most common drug metabolising enzymes were selected. Incubation of CCT241736 with individual rCYPs revealed CYP3A4 to be the predominant enzyme in the metabolism of this compound, responsible for formation of five out of seven observed metabolites (M1, M2, M4, M5 and M7) and postulated to be involved in the formation of a sixth (M6). Indeed, the scaled CL_int_ of CCT241736 in rCYP3A4 exceeded that observed in HLM. Whilst discrepancies in the total clearance of a compound between such systems are not unexpected due to inaccuracy in the ISEF value and/or protein binding, this result confirms CYP3A4 as the major contributor to oxidative metabolism of CCT241736. Consequently, co-administration of CCT241736 and CYP3A4 inhibitors (such as the azole antifungals often used prophylactically in cancer treatment (Cronin and Chandrasekar, 2010)) is therefore likely to be contraindicated. CYP2C9 and CYP2D6 were the only other CYPs found to metabolise CCT241736; M3 was identified in incubations with each of these enzymes. The absence of M6 from any individual rCYP incubation implies the involvement of multiple enzymes in its production and, owing to the structural similarities between these metabolites, M6 is postulated to be formed by secondary metabolism of M1 and/or M3. Yet, as this has not been experimentally determined, the conversion of M1 to M6 and M3 to M6 by CYP2C9/2D6 and CYP3A4 respectively is asterisked and accompanied by dashed arrows in Fig. 4. M7 is also believed to be a secondary metabolite, although only CYP3A4/5 are implicated in its production. This notion is based on the profile of M7 in mouse (undetectable until 15 min, but increasing subsequently) and its structural similarity to both M1 and M4. Indeed, the profiles of M1 and M4 in MLM suggest that both undergo further metabolism (Fig. 3B). The presence of M7 in HLM indicates that CCT241736 follows the same or a similar metabolic pathway in human as in mouse; its apparent absence in human hepatocytes is likely a consequence of its very low clearance coupled with analytical limitations of sensitivity. M7 is therefore proposed as a secondary metabolite of M1 and/or M4 in human, but as for M6, these pathways are represented by dashed arrows to highlight this uncertainty. Since it was concluded that all seven metabolites previously identified in human hepatocytes and HLM were plausibly formed by one or more rCYPs, it was deemed that no further investigation of reaction phenotyping was required.

**Fig. 5:**
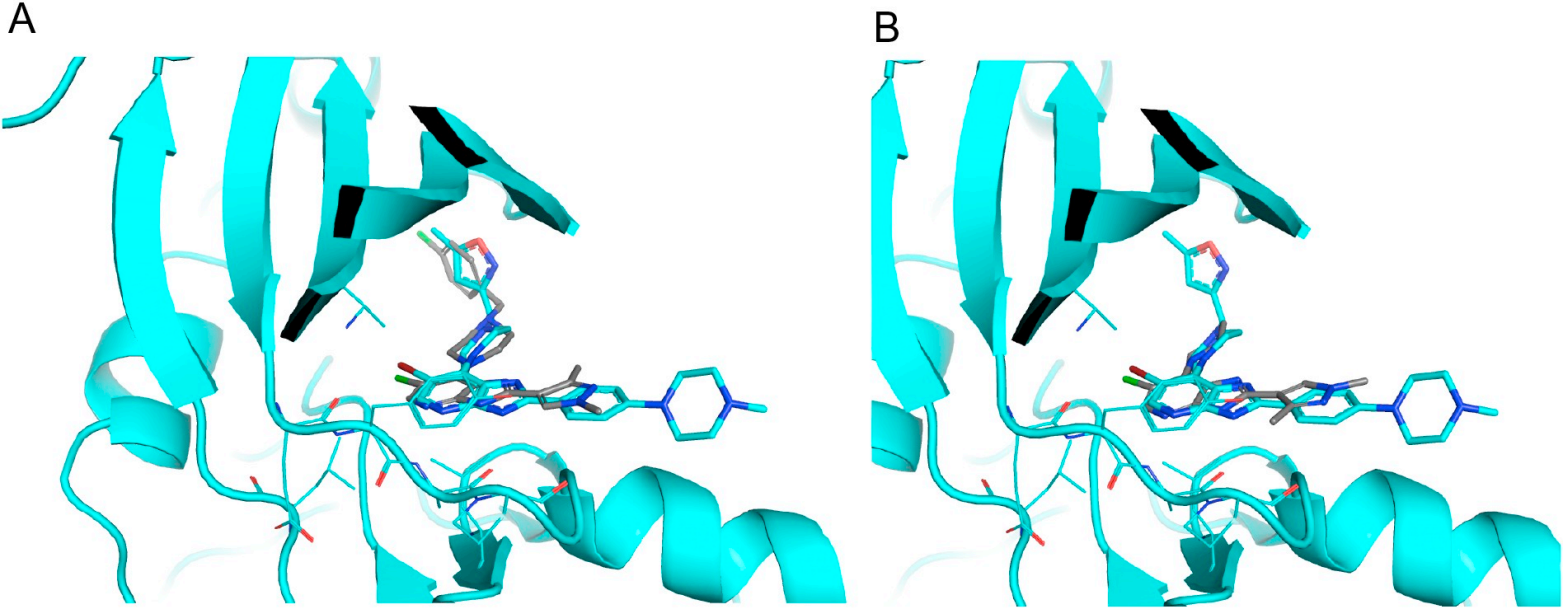
Predicted binding mode of CCT241736 (dark grey) (A) and M1 (dark grey) (B) overlaid with the bound inhibitor compound 51 (cyan) in Aurora-A crystal structure (PBD 2x6e).

As the most abundant metabolite in human *in vitro* systems (maximally approximately 10% of T_0_ parent based on LC-MS response in both hepatocytes and liver microsomes), it was prudent to test the activity of M1 against the targets of the parent compound (Aurora kinases A and B, FLT3 and FLT3-ITD). M1 was prepared by large scale incubation of CCT241736 with rCYP3A4 followed by separation by HPLC and sample concentration. Importantly, M1 was found to retain activity against FLT3 (K_d_ = 7.9 nM, compared to 6.2/26 nM for parent), but some drop-off was seen for Aurora-A, Aurora-B and FLT3-ITD (Table 3). The reduced potency for Aurora-A is consistent with the predicted binding pose of M1 obtained from the molecular docking study (Fig. 5). Whilst this metabolite is not expected to exceed 10% of circulating levels of parent *in vivo* and therefore does not warrant additional toxicological testing, such information may be a useful input into pharmacokinetic-pharmacodynamic (PKPD) modelling of the parent compound.

In conclusion, *in vitro* assessment of the metabolism of CCT241736 in human and four pre-clinical species was undertaken. Based on the metabolic information, selection of minipig as the larger mammalian species for toxicology studies is justified. In addition, the minipig also shows greater similarities to human than the dog in both intestinal and heart physiology and electrophysiology (Singh et al., 2016), therefore increasing the likelihood that any adverse effects relating to these systems in human will be identified prior to administration. The metabolic profile of CCT241736 in mouse and rat was sufficiently similar to human for either to be an appropriate toxicology species, albeit CL_int_ was higher in mouse. In light of the increased data available for this compound from earlier PKPD and therapy studies in human tumour xenograft mouse models, mouse was selected as the rodent toxicology species. All human metabolites of CCT241736 are believed to be produced by CYPs, CYP3A4 being the major enzyme responsible for metabolism of this compound. The most abundant human metabolite *in vitro* (M1) was demonstrated to retain activity against FLT3, but exhibited some loss of activity against Aurora-A, as well as Aurora-B and FLT3-ITD. This study provides valuable information to support the further progression of CCT241736 as a candidate for the treatment of AML.

The following are the supplementary data related to this article.

Table 1 Full MS/dd-MS^2^ and Full MS/AIF workflow parameters. Table 1

**Fig. 1:**
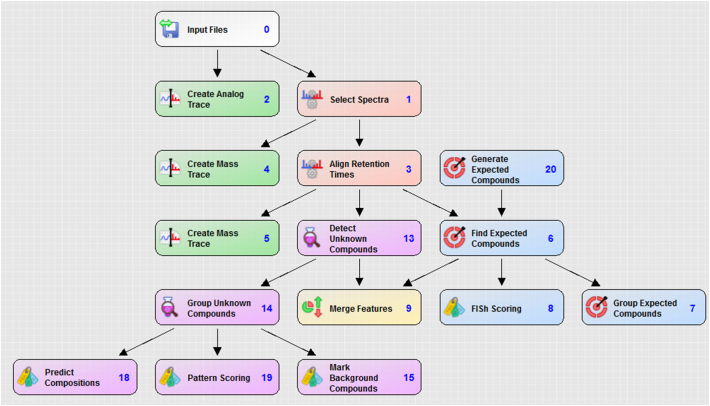
Compound Discoverer targeted workflow for analysis of LC-MS/MS data.

Fig. 2 Fragmentation spectra of parent (A), M2 (B) and M5 (C) in HLM sampled at 60 min and M8 (D) in male dog hepatocytes sampled at 120 min. Fig. 2

## Funding

This work was supported by Cancer Research UK (grant number C309/A11566).

## Declarations of interest

None.
