# Supplementary material for: Metabolism of the dual FLT-3/Aurora kinase inhibitor CCT241736 in preclinical and human in vitro models: Implication for the choice of toxicology species
Source: Eur J Pharm Sci. 2019 Nov 1;139:104899. doi: 10.1016/j.ejps.2019.04.004 (PMC6892276; doi:10.1016/j.ejps.2019.04.004)
Supplement: Table 1 — Full MS/dd-MS2 and Full MS/AIF workflow parameters. [file mmc1.doc]

**Appendix**

| **Workflow** | | | |
| --- | --- | --- | --- |
| **Full MS/dd-MS^2^** | | **Full MS/AIF** | |
| Resolution  AGC Target  Max Inject Time (ms)  Scan Range (m/z) | 70,000  1e6  100  120-1200 | Resolution  AGC Target  Max Inject Time (ms)  Scan Range (m/z) | 70,000  1e6  200  80-1200 |
| **dd-MS^2^** | |  |  |
| Resolution  AGC Target  Max Inject Time (ms)  Minimum AGC Target | 17,500  1e5  50  8e3 |  |  |

**Table 1. Full MS/dd-MS^2^ and Full MS/AIF workflow parameters.**
