# Supplementary material for: Metabolism of the dual FLT-3/Aurora kinase inhibitor CCT241736 in preclinical and human in vitro models: Implication for the choice of toxicology species
Source: Eur J Pharm Sci. 2019 Nov 1;139:104899. doi: 10.1016/j.ejps.2019.04.004 (PMC6892276; doi:10.1016/j.ejps.2019.04.004)
Supplement: Fig. 2 — Fragmentation spectra of parent (A), M2 (B) and M5 (C) in HLM sampled at 60 min and M8 (D) in male dog hepatocytes sampled at 120 min. [file mmc2.docx]

**A****

| m/z | Formula | Structure |
| --- | --- | --- |
| 456.14636 | C_22_H_24_N_7_Cl_2_ |  |
| 330.12274 | C_15_H_17_N_7_Cl |  |
| 289.09644 | C_13_H_14_N_6_Cl |  |
| 262.07297 | C_11_H_11_N_6_Cl |  |
| 168.05785 | C_9_H_11_NCl |  |
| 125.01560 | C_7_H_6_Cl |  |

**B**

| m/z | Formula | Structure |
| --- | --- | --- |
| 472.14316 | C_22_H_24_ON_7_Cl_2_ |  |
| 330.12308 | C_15_H_17_N_7_Cl |  |
| 289.09628 | C_13_H_14_N_6_Cl |  |
| 275.08069 | C_12_H_12_N_6_Cl |  |
| 125.01560 | C_7_H_6_Cl |  |
| 83.06107 | C_4_H_7_N_2_ |  |

**C**

| m/z | Formula | Structure |
| --- | --- | --- |
| 472.14197 | C_22_H_24_ON_7_Cl_2_ |  |
| 330.12204 | C_15_H_17_N_7_Cl |  |
| 289.09637 | C_13_H_14_N_6_Cl |  |
| 275.08069 | C_12_H_12_N_6_Cl |  |
| 184.05252 | C_9_H_11_ONCl |  |
| 141.01031 | C_7_H_6_OCl |  |

**D**

| m/z | Formula | Structure |
| --- | --- | --- |
| 458.12613 | C_21_H_22_ON_7_Cl_2_ |  |
| 440.11548 | C_21_H_20_N_7_Cl_2_ |  |
| 332.10205 | C_14_H_15_ON_7_Cl |  |
| 291.07593 | C_12_H_12_ON_6_Cl |  |
| 273.06519 | C_12_H_10_N_6_Cl |  |
| 259.04922 | C11H8N6Cl |  |
| 168.05785 | C_9_H_11_NCl |  |
| 125.01560 | C_7_H_6_Cl |  |
